# Systems genetics identifies methionine as a high risk factor for Alzheimer's disease

**DOI:** 10.3389/fnins.2024.1381889

**Published:** 2024-07-16

**Authors:** Congmin Wang, Yu Hei, Yu Liu, Akhilesh Kumar Bajpai, Yuhe Li, Yawen Guan, Fuyi Xu, Cuifang Yao

**Affiliations:** ^1^School of Pharmacy, Binzhou Medical University, Yantai, China; ^2^Shandong Technology Innovation Center of Molecular Targeting and Intelligent Diagnosis and Treatment, Binzhou Medical University, Yantai, China; ^3^Department of Genetics, Genomics and Informatics, University of Tennessee Health Science Center, Memphis, TN, United States

**Keywords:** Alzheimer's disease, methionine, GSK-3β, dopaminergic synapse, GeneNetwork

## Abstract

As a dietary strategy, methionine restriction has been reported to promote longevity and regulate metabolic disorders. However, the role and possible regulatory mechanisms underlying methionine in neurodegenerative diseases such as Alzheimer's disease (AD), remain unexplored. This study utilized the data from BXD recombinant inbred (RI) mice to establish a correlation between the AD phenotype in mice and methionine level. Gene enrichment analysis indicated that the genes associated with the concentration of methionine in the midbrain are involved in the dopaminergic synaptic signaling pathway. Protein interaction network analysis revealed that glycogen synthase kinase 3 beta (GSK-3β) was a key regulator of the dopaminergic synaptic pathway and its expression level was significantly correlated with the AD phenotype. Finally, *in vitro* experiments demonstrated that methionine deprivation could reduce the expression of Aβ and phosphorylated Tau, suggesting that lowering methionine levels in humans may be a preventive or therapeutic strategy for AD. In conclusion, our findings support that methionine is a high risk factor for AD. These findings predict potential regulatory network, theoretically supporting methionine restriction to prevent AD.

## 1 Introduction

Alzheimer's disease (AD), the main cause of dementia (Jorfi et al., 2023), is characterized by progressive neurodegeneration (De Strooper and Karran, 2016). According to the Alzheimer's Disease International Organization, there are ~9.5 million patients with dementia in China, and it is projected to exceed 16 million by 2030 (Jia et al., 2020). Patients with AD experience memory loss, cognitive decline (Qu et al., 2022), disorientation (Atri, 2019), impaired judgment, and sometimes hallucinations or delusions (Knopman and Petersen, 2014), significantly affecting their independence and quality of life. Although there is currently no approved cure for AD (Gong et al., 2019), pharmacological and non-pharmacological therapies can help improve patients' quality of life and slow down disease progression (Zhang et al., 2013; Kong et al., 2020; Thota et al., 2020; Xu et al., 2023). Several pathophysiological factors have been implicated in the atrophy of the cerebral cortex and hippocampus (Li Y. D. et al., 2023), including abnormalities in neurotransmitters, beta-amyloid-induced plaque deposition, neurofibrillary tangle formation, inflammation (Li et al., 2019), and oxidative stress (Chen et al., 2021; Pan et al., 2021; Yang et al., 2023). Accumulation of hyperphosphorylated Tau protein disrupts synaptic function and leads to brain dysfunction by impairing glutamate receptor trafficking or synaptic anchoring (Miller et al., 2014; Tanaka et al., 2022). Furthermore, there is a correlation between Tau hyperphosphorylation and the tangles of AD (Guillozet-Bongaarts et al., 2006; Giovinazzo et al., 2021; John and Reddy, 2021). Although the triggers, underlying mechanisms, and effective treatments of AD are still unclear, recent studies have shown that dietary interventions may be potential treatment strategies (Valls-Pedret et al., 2015). Dietary restriction during adulthood (DR; reducing calorie intake while maintaining micronutrient levels) reduces the risk of AD (Nuru et al., 2018; Kovalska et al., 2023). Age-related impairments in learning, memory, and motor function were observed to improve in rodents undergoing dietary restriction, however the mechanics remain unclear (Xu et al., 2022).

Recently researchers have found that amino acid homeostasis is disrupted in the serum and brain of patients with AD (Trushina et al., 2013; Lin et al., 2018; Wang et al., 2021; Li et al., 2022; Zinellu et al., 2023). Moreover, alterations in the levels of different amino acids in the physiological range have been linked to various pathological conditions, including neurological disorders (Kepka et al., 2020). Longitudinal studies using mouse models of AD have also demonstrated abnormal essential amino acid levels (Puris et al., 2023). These findings suggest that dietary intervention may affect the progression of AD by regulating amino acids metabolism.

Methionine is a widely-used sulfur-containing amino acid that serves as a precursor for substances such as spermine, spermidine, and ethylene (Parkhitko et al., 2019). It plays a pivotal role in various aspects of growth and development (Lu et al., 2001), including cell division (Yang et al., 2022), differentiation, apoptosis, homeostasis, and gene expression (Martínez-Chantar et al., 2008; Li M. L. et al., 2023). Studies have shown that the methionine cycle is involved in the pathogenesis of AD (Zhao et al., 2022). Methionine serves as a crucial methyl donor in certain methyltransferase reactions, providing methyl groups to various compounds. High methionine diet has been proven to induce AD-like symptoms (Pi et al., 2021). As a dietary intervention, methionine restriction has been reported to alleviate AD, but the molecular mechanisms remain unclear (Alachkar et al., 2022; Kovalska et al., 2023; Xi et al., 2023). Therefore, it is of great significance to explore the specific mechanisms, by which methionine is involved in the pathogenesis of AD.

The systems genetics approach serves as a robust instrument relying on systems biology and mouse genetic reference panel (Civelek and Lusis, 2014). BXD recombinant-inbred (RI) mice derived from the crosses between C57BL/6J (B6) and DBA/2J (D2) inbred strains, is a large and well-characterized genetic reference population, and has been widely used for studying the genetic basis of various diseases (Ashbrook et al., 2021). Thousands of transcriptomic/phenomic datasets have been published using these BXD strains over the past decades, consisting of ~250 phenotypes associated with cognitive function (Philip et al., 2010). In addition, since each BXD strain is a stable inbred, it can be replicated in large numbers, to facilitate the precise localization of complex traits with low to moderate heritability (Xu et al., 2024). The BXD panel showed a strong variation in methionine concentrations in the midbrain across different strains, suggesting that it is a suitable population for exploring potential mechanisms underlying the association between methionine and AD.

In the present study, we first investigated the associations between methionine level and AD. We then verified the correlation in SH-SY5Y cell lines. Further analysis based on the BXD RI mice database unveiled that the dopaminergic synaptic signaling pathway may play a key role in the regulation of AD through methionine. The key regulator was predicted to be glycogen synthase kinase 3 beta (GSK-3β). Our study revealed a positive association between methionine level and increased risk of AD. However, it is necessary to validate this predictive regulatory mechanism *in vivo* and *in vitro*.

## 2 Materials and methods

### 2.1 BXD midbrain transcriptomic data set

The mRNA expression data set, VU BXD Midbrain Agilent SurePrint G3 Mouse GE (May12) was obtained from the GeneNetwork website (https://www.genenetwork.org/) (Xu et al., 2011). The expression data was generated from the adult male mice of 34 different BXD strains (Xu et al., 2020) between 4 and 12 months of age. They were housed in groups in a vivarium that controlled temperature and humidity levels. Additionally, the mice were subjected to a 12-h light-dark cycle. The mice had unrestricted access to both food and water. The array data in GeneNetwork have been log transformed and then the z-score normalized, where instead of leaving the mean at 0 and the standard deviation of 1 unit, the data is rescaled to a mean of 8 units with a standard deviation of 2 units (what we call 2Z + 8 normalized data). The standardized midbrain methionine level dataset is available on GeneNetwork, belonging to the group “BXD Family,” categorized as “Phenotypes,” and named “18038.”

### 2.2 Behavioral phenotypes access

The learning-related traits of BXD mice were retrieved from GeneNetwork. The detailed descriptions of the traits can be found in GeneNetwork and in a previous publication (Neuner et al., 2019a). The summary statistics and individual values are available under the “BXD” group, “trait and cofactors” type, and “BXD phenotypes” data set with record IDs of 20494, 20499, 20575, and 20817.

### 2.3 Gene-phenotype correlation analysis

Gene-phenotype correlation analysis has been widely used to investigate key genes in a gene set. The gene-phenotype correlations were performed on the GeneNetwork online platform using Pearson's correlation method. We used this method to investigate the correlated genes in BXD mouse midbrain transcriptomic dataset with the midbrain methionine level phenotype (ID: 18038). Genes with *p*-values < 0.05 were considered to be significantly associated with the 18038 phenotype.

### 2.4 Pathway enrichment analysis of co-expressed genes

The pathway database is a collection of manually created Kyoto Encyclopedia of Genes and Genomes (KEGG) pathway maps that represent the molecular wiring diagrams of biological systems (Deng et al., 2021). Genes with statistically significant genetic correlation with 18038 phenotype (*p* < 0.05) were selected and uploaded to WebGestalt (http://www.webgestalt.org/) for gene enrichment analysis. This analysis employs a hypergeometric statistical test to generate adjusted *p*-values and enrichment ratios. The *p*-values generated from the hypergeometric test were corrected by the Benjamini-Hochberg method. The pathways with adjusted *p* < 0.05 were considered significant.

### 2.5 Protein-protein interaction (PPI) network analysis

PPI networks provide useful information about cellular functions and biological processes. The gene set was submitted to the online tool STRING (Szklarczyk et al., 2019), a database of known and predicted PPIs (https://string-db.org/). We explored PPI networks for genes in the dopaminergic pathway in the STRING database.

### 2.6 Gene-phenotype network analysis

The gene-phenotype network analysis was performed on the GeneNetwork online platform using Pearson's correlation. Using the BXD midbrain transcriptomic dataset, we deployed a gene-phenotype expression network to identify the key genes from the gene set as previously described (Deng et al., 2021). Briefly, a network was constructed using Pearson's correlation coefficient matrix. In the network, each node stood for a gene, and the correlation coefficient was set as the edge. Binomial correlations higher than 0.3 or lower than −0.3 were defined as connected. The connection weight was calculated for each node as, the sum of the binominal correlation coefficient connected to each node. The correlation coefficients with *p*-values < 0.05 were deemed statistically significant.

### 2.7 Cell culture

The SH-SY5Y cell line, derived from human neuroblastoma, was obtained from ATCC and was authenticated by short-tandem repeat profiling. These neuronal precursor cells were cultured in MEM-F12 medium supplemented with 10% fetal bovine serum and 1% penicillin-streptomycin solution. The cells were maintained at a temperature of 37°C and a carbon dioxide (CO_2_) concentration of 5%. The cells were treated with the GSK-3β inhibitor (CHIR-99021, APE × BIO) with a final concentration of 8 μM to further study.

### 2.8 Western blotting

To prepare the samples for analysis, a loading buffer was added to cells. They were then subjected to boiling for 10 min. Following boiling, centrifugation was performed at 12,000 rpm/min for 5 min to separate the samples. The resulting samples were loaded onto a 10%-12% SDS-PAGE gel and subsequently transferred onto a polyvinylidene fluoride (PVDF) membrane using a wet transfer apparatus. To block non-specific binding, the membrane was incubated with 5% skimmed milk or BSA for 1 h. After blocking, the membrane was washed three times with TBST (Tris-buffered saline with Tween 20), for 10 min each. The membrane was then incubated with the designated primary antibody at 4°C overnight. Thereafter, the secondary antibody was added and incubated for 1 h. Finally, luminescence was performed to visualize the protein bands. An imaging system and ImageJ software were used to quantify the protein bands. These tools allowed for the accurate measurement and analysis of the protein bands. The following primary antibodies were used for Western blotting: mouse anti-actin (1:2,000 dilution, GenScript, A700702), rabbit anti-Tau (1:2,000 dilution, proteintech, 10274-1-AP), mouse anti-p-Tau (1:2,000 dilution, Santa Cruz, sc-32275), rabbit anti-Aβ (1:1,000 dilution, CST, 8243T), mouse anti-GSK3 beta (1:1,000 dilution, Santa Cruz, sc-81462), and mouse anti-p-GSK3 alpha/beta (1:1,000 dilution, Santa Cruz, sc-81496).

### 2.9 Statistical analysis

Data were analyzed using GraphPad Prism software. The results are presented as mean ± SD. Comparisons between the two groups were evaluated using two-tailed Student's *t*-test to determine significant *p*-values. ^*^p < 0.05, ^**^p < 0.01, and ^***^p < 0.001 were considered statistically significant.

## 3 Results

### 3.1 The levels of methionine exhibited variability across BXD strains

**Figure 1:**
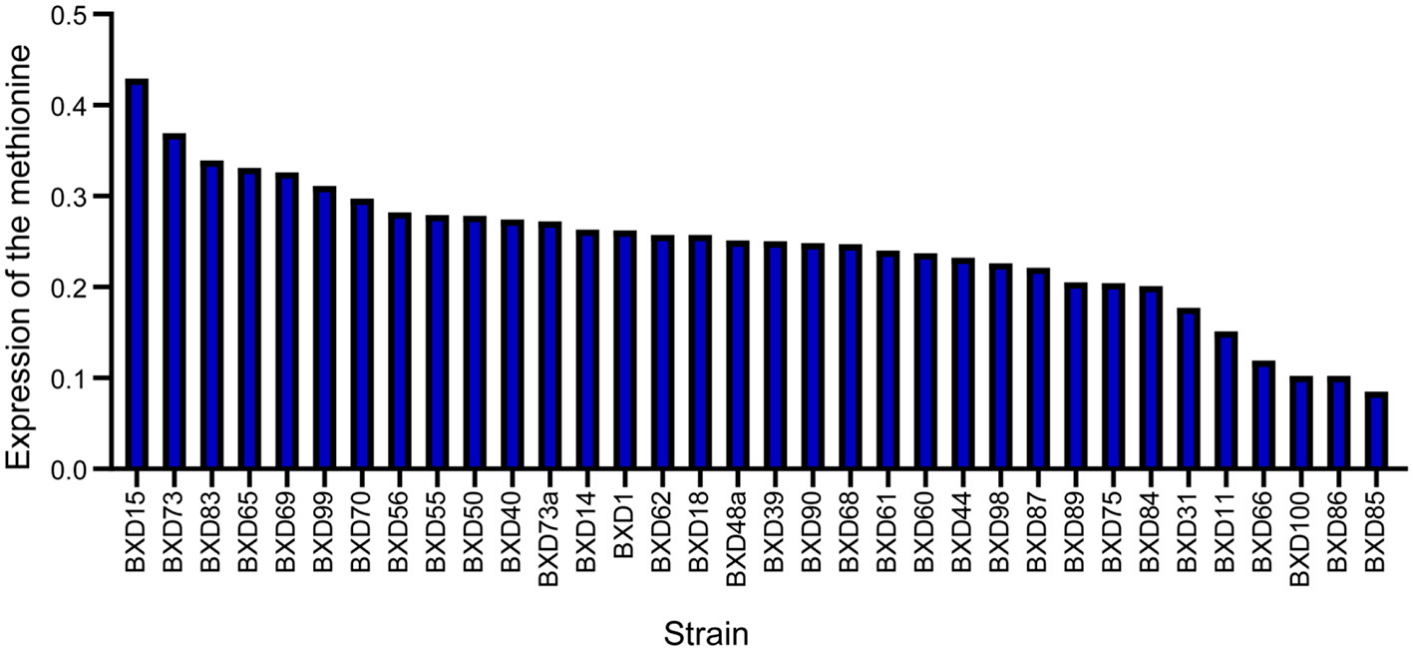
Expression of methionine in the BXD family. Bar-plot of methionine expression level in the midbrain across BXD strains. BXD15 shows the highest expression and BXD85 shows the lowest expression. The x-axis shows BXD strains. The y-axis shows the normalized log2 expression levels of methionine.

To determine whether methionine regulates AD, systems genetics analysis was conducted to uncover the expression regulation of methionine levels in the midbrain. Although AD has been mainly studied in the context of hippocampus, a relationship has also been reported between the midbrain and AD. Methionine levels were examined in the midbrain of 34 BXD mice strains in GeneNetwork. The midbrain transcriptome data of BXD RI mice used are available through our GeneNetwork website. The standardized dataset “midbrain methionine level” is available on GeneNetwork (group “BXD Family,” category “Phenotypes,” named “18038”). There were a significant differences in methionine levels among the BXD strains with thresholds ranging from 0.4 (BXD15) to 0.09 (BXD85). The average expression threshold was 0.25, while the median expression threshold was 0.25. Further, we observed a 4.4-fold change in methionine expression levels across the 34 BXD strains (Figure 1). These data were utilized for conducting association analyses with AD phenotypes.

### 3.2 Midbrain methionine levels were linked to AD

**Figure 2:**
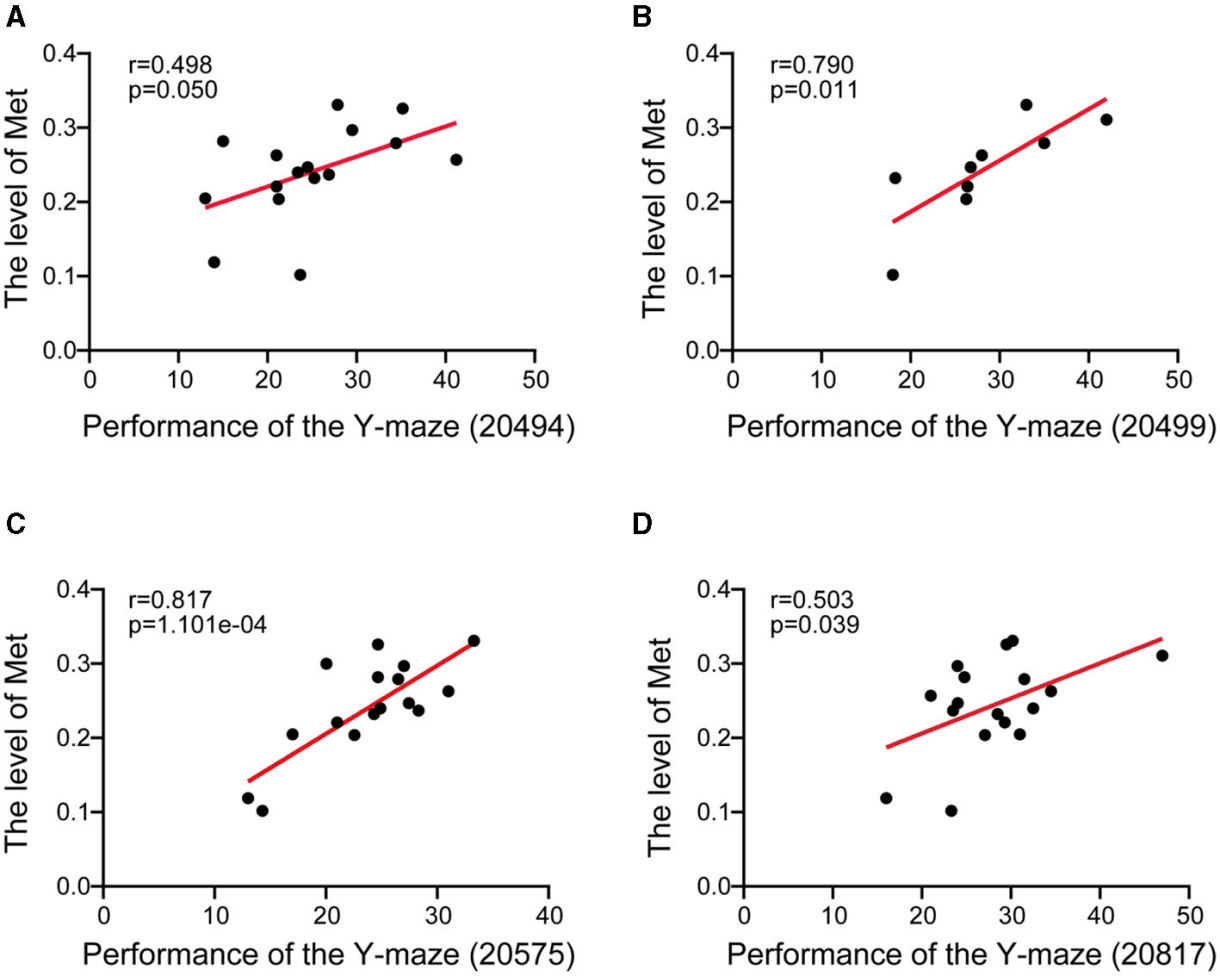
Scatter plot showed the correlation between midbrain methionine level and the AD-related behavioral phenotypes. **(A–D)** The Pearson's correlation coefficient was used to determine the relationship. **(A–D)** The correlations between methionine levels (log2 transformed) in the midbrain and the percentage of unsuccessful alternations in the Y-maze test in BXD mice [**(A)**: Record ID 20494; **(B)**: Record ID 20499; **(C)**: Record ID 20575; **(D)**: Record ID 20817]. Pearson correlation coefficients and *p*-values are indicated.

The Y-maze method is a valuable tool for evaluating both conditioned reflexes and spatial memory in animals. The Y-maze is not only used for assessing spatial working and reference memory in the hippocampus but also commonly used to detect the function of learning and memory in the midbrain (Ishola et al., 2018, 2019; Bashirzade et al., 2022). To investigate the effect of methionine on memory and cognitive performance, we conducted Pearson's correlations between methionine level and Y-maze performance phenotypes. The methionine level was found to be significantly positively correlated with the percentage of unsuccessful alternations in the Y-maze test [*n* = 16, *r* = 0.498, *p* = 0.050 (Figure 2A), *n* = 9, *r* = 0.790, *p* = 0.011 (Figure 2B), *n* = 16, *r* = 0.817, *p* =0.0001101 (Figure 2C), and *n* = 17, *r* = 0.503, *p* = 0.039 (Figure 2D)], suggesting that methionine concentration positively affects the course of AD.

### 3.3 GSK-3β mediates methionine-induced AD through the dopamine signaling pathway

**Figure 3:**
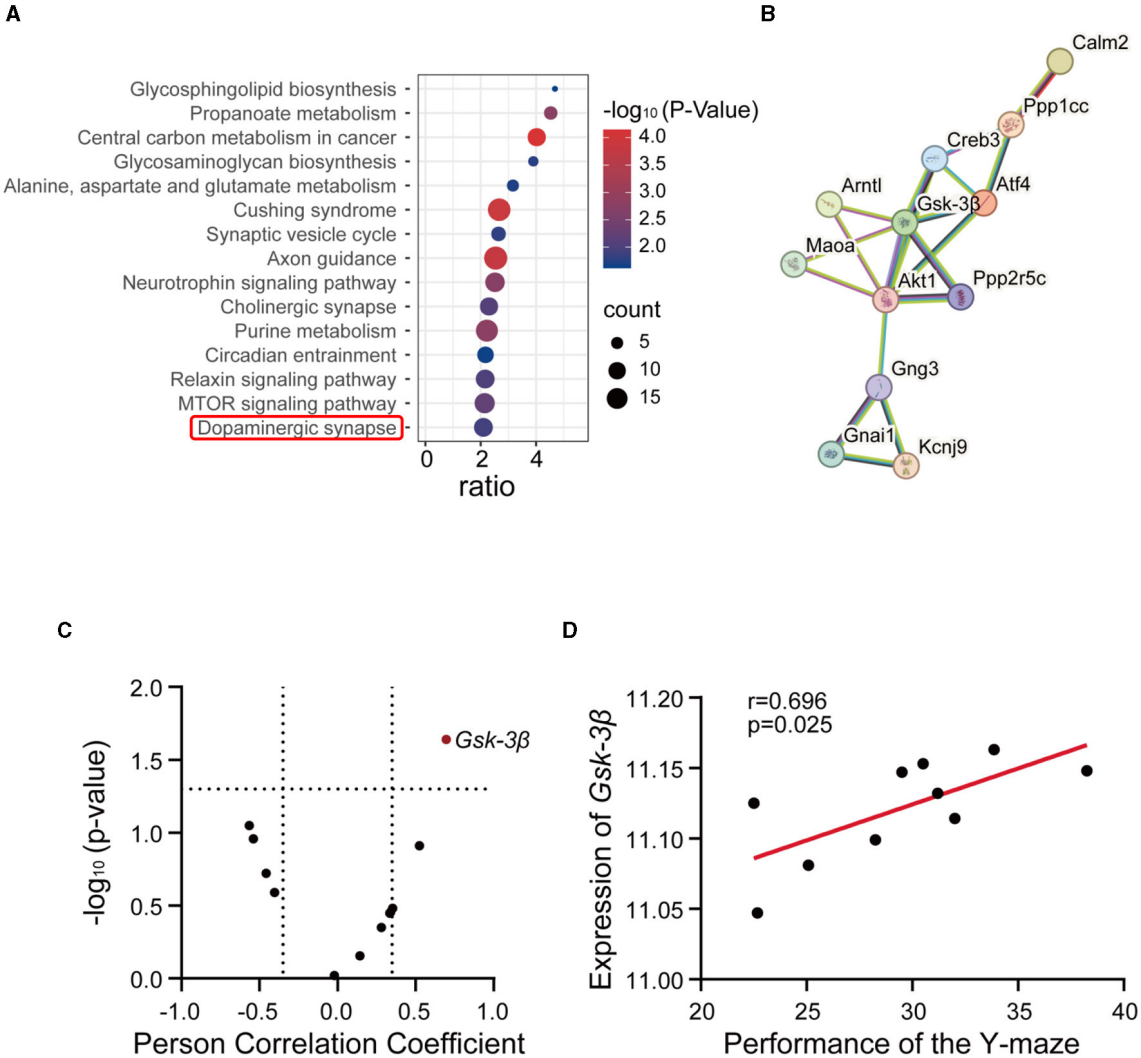
GSK-3β was associated with methionine-induced cognitive impairment. **(A)** The bubble plot showed the KEGG pathways significantly enriched by the methionine-correlated genes in the midbrain of BXD mice. The analysis was performed using WebGestalt (http://www.webgestalt.org/), and significant genes were identified (*p* < 0.05). The x-axis represents the enrichment ratio, while the y-axis represents the enriched pathways. The size of the bubbles corresponds to the number of genes, and the color represents the *p*-value. **(B)** Protein-protein interaction network of dopaminergic pathway genes. In total, 12 dopaminergic pathway genes were submitted to the STRING database (https://cn.string-db.org/). **(C)** The correlations between all genes of the dopaminergic pathway in the midbrain and the percentage of unsuccessful alternations in the Y-maze test in BXD mice (Record ID 20565). Red dots represent significant positive associations (*p* < 0.05). **(D)** The correlations between the expression of GSK-3β in the midbrain and the percentage of unsuccessful alternations in the Y-maze test in BXD mice (Record ID 20565).

To unravel the role of methionine in AD, we performed gene-set enrichment analysis using the top 2,000 genes correlated with methionine level in the midbrain (*p* < 0.05, Pearson's correlation; Supplementary Table S1). We performed KEGG pathway enrichment analysis and selected the top 15 pathways for further analysis (Supplementary Table S2). The analysis indicated the enrichment of neurodegenerative disease-associated pathways, such as synaptic vesicle cycle, axon guidance, cholinergic synapse, circadian entrainment, and dopaminergic synapse (Figure 3A). Among these, the dopaminergic synapse pathway exhibited a strong association with the AD phenotypes. Dysfunction or abnormalities in dopaminergic synapses have been implicated in various neurological and psychiatric disorders, including Parkinson's disease, schizophrenia, and addiction (Song et al., 2023). To identify the specific mechanisms, we subjected the dopamine pathway genes to protein interaction analysis based on the STRING database (https://cn.string-db.org/). The PPI analysis revealed *GSK-3*β to be at the center of the network (Figure 3B). GSK-3β has been known to play a fundamental role in various processes, including cell division, proliferation, differentiation, and adhesion (Forde and Dale, 2007). Moreover, it has been implicated in various disorders, including AD and hyperdopamine dependent behaviors (Beaulieu et al., 2007; Lauretti et al., 2020). Using the percentage of unsuccessful alternations in the Y-maze test, we conducted a Pearson's correlation correlation analysis of the dopaminergic pathway genes in GeneNetwork to investigate the relevance of the dopaminergic pathway in AD. The results showed a strong positive correlation between *GSK-3*β and Y-maze phenotype (Figures 3C, D), demonstrating a positive association of *GSK-3*β expression with the progression of AD. These results further suggest that methionine may promote AD progression by positively regulating *GSK-3*β.

### 3.4 Methionine deficiency alleviated the progression of AD

**Figure 4:**
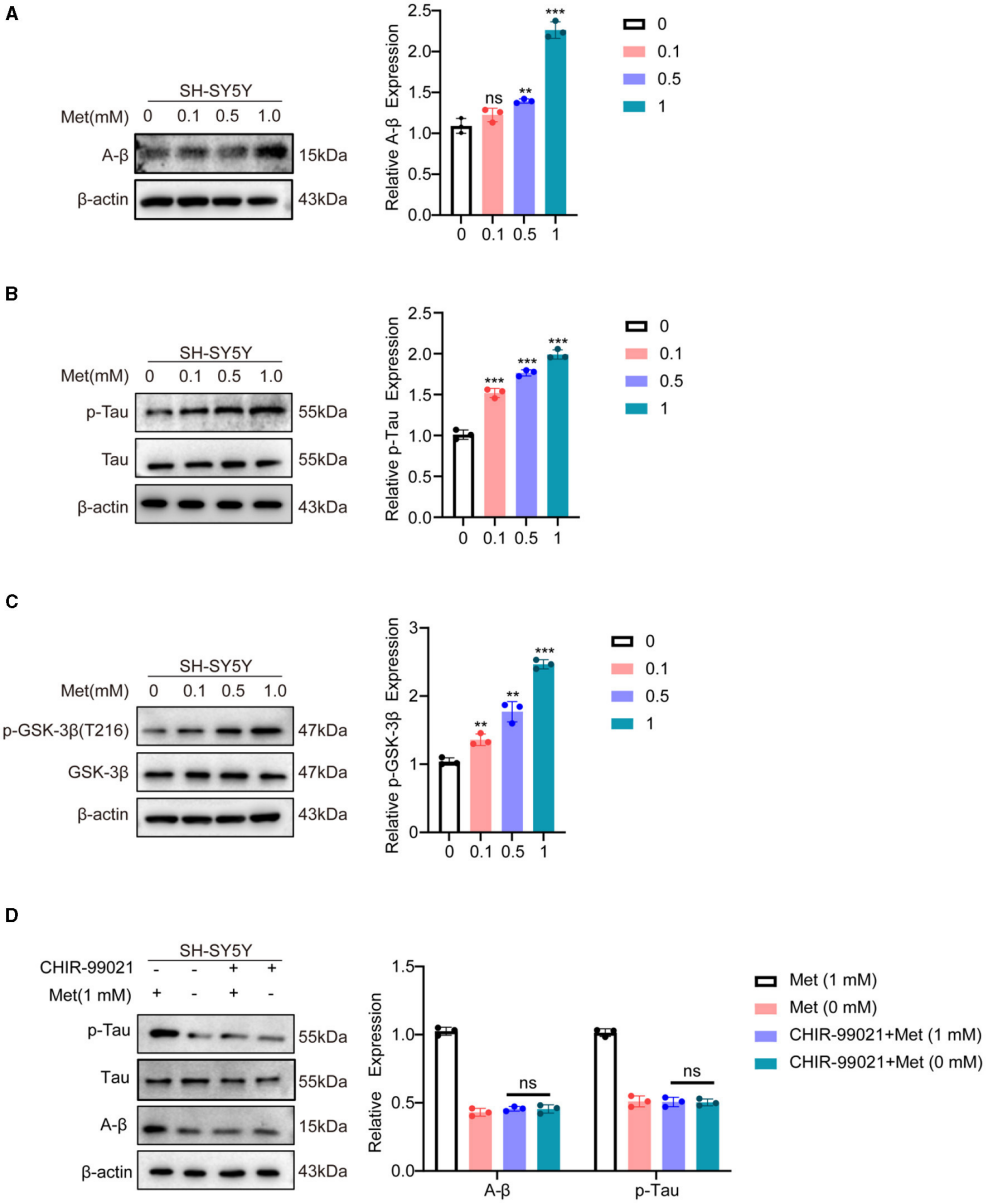
Methionine deficiency inhibited the progression of AD. SH-SY5Y cells were treated with methionine at different concentrations. **(A, B)** Phosphorylation of Tau and A-β, as typical pathological features of AD, was detected using Western blotting after treatment with different concentrations of methionine. **(C)** After treatment with different concentrations of methionine, GSK-3β phosphorylation was detected using Western blotting. **(D)**. Changes in A-β and p-Tau protein levels using co-treatment with GSK-3β inhibitor and different concentrations of methionine.

In the central nervous system, two secretory enzymes convert amyloid precursor protein (APP) generates amyloid β (Aβ) (Soria Lopez et al., 2019). The Tau protein is primarily expressed in neurons and plays a crucial role in microtubule protein polymerization and microtubule stabilization (Ossenkoppele et al., 2022). Aβ induces Tau hyperphosphorylation, oxidative stress, and inflammatory response, leading to cell death and impairing neurotransmission. AD is diagnosed based on the presence of Aβ and phosphorylated Tau. Our results demonstrated a dose-dependent upregulation of Aβ expression with increasing methionine concentrations (Figure 4A). Phosphorylation levels of Tau protein were also found to be elevated after treatment with high concentrations of methionine (Figure 4B). GSK-3β is a multifunctional protein and plays a crucial role in the pathogenesis of AD as a small molecule kinase (Hurtado et al., 2012). Inhibiting GSK-3β activity has been shown to improve cognitive impairments and attenuate oxidative stress. It has been reported that AD brains possess high levels of GSK-3β (Farr et al., 2014). Relevant studies have shown that phosphorylation of the Tyr216 site of GSK-3β can promote its activation, hyperphosphorylate Tau, activate the Thr668 site of APP protein, and induce pathological changes in AD. Phosphorylation by activated Akt at the Ser9 site, inactivates GSK-3β (Steen et al., 2005; Martin et al., 2011; Yang et al., 2020). Our findings indicated that phosphorylation of the Tyr216 site of GSK-3β is methionine-dependent, and high concentrations of methionine promote its expression (Figure 4C). To further demonstrate whether methionine regulates AD progression by modulating GSK-3β, the results of our experiments revealed that methionine deficiency or excess no longer altered A-β and p-Tau levels when we treated SH-SY5Y cells with the GSK-3β inhibitor, the above results proved our speculation (Figure 4D). However, the mechanism of how methionine regulates GSK-3β in AD still needs further studies.

## 4 Discussion

In this study, we found that high levels of methionine were positively associated with the percentage of unsuccessful alternations in the Y-maze test which represents more severe learning and memory dysfunction. We then treated SH-SY5Y cells with different concentrations of methionine and found that Aβ and p-Tau levels were positively correlated with methionine levels. High methionine levels in the medium significantly upregulated Aβ and p-Tau. Removal of methionine from the medium significantly decreased Aβ and p-Tau levels. These results support methionine as a high risk factor for AD. These findings are also consistent with previous reports that dietary restriction of methionine can improve the symptoms of AD (Xi et al., 2023).

Non-pharmacological treatments are valuable for preventing AD or complementing other treatments. Dietary changes in many metabolites have shown clear preclinical benefits, some of which have also shown promise in clinical trials. Although the effect of dietary interventions on the progression of AD is well-known, the previous studies are still scarce and controversial. There are still no clear guidelines or recommended dietary change programs for patients with AD. Some studies have found that the Mediterranean diet, the Dietary Approaches to Stop Hypertension (DASH), and the Mediterranean-DASH Intervention for Neurodegenerative Delay diet (MIND), may protect against AD (van den Brink et al., 2019). Many potential dietary factors have been implicated in the development of AD, including dietary deficiencies of folic acid and vitamins E, C, B6, and B12 (Luchsinger and Mayeux, 2004; An et al., 2019; Mielech et al., 2020). Antioxidant vitamins affect lipid peroxidation and oxidative stress. Folic acid and vitamins B6 and B12 regulate DNA methylation, which can increase homocysteine levels. It has also been reported that excess saturated fatty acids may be a potential risk factor for AD (Luchsinger and Mayeux, 2004). However, there are few reports about the role of a single amino acid on AD. Previous studies have suggested that impaired BCAA metabolism can impact neuronal health and synaptic function, potentially affecting the development and progression of neurodegenerative disorders (Wang et al., 2023). More studies are needed to support the regulatory role of a single amino acid in AD.

In this study, we integrated phenotypic resources from the BXD panel, including more than 300 neural phenotypes from over 150 mouse strains. The recombinant inbred population of the BXD family, as one of the largest and well-characterized genetic reference populations, is the basis for studying various diseases, such as cancer, cognitive disorders, and heart diseases (Zhu et al., 2020; Bajpai et al., 2023; Zhou et al., 2023; Xu et al., 2024). The correlation of the BXD RI family has been extended to genetic analysis of behavioral phenotypes, including drug addiction, neurodegenerative processes, etc. (Wang et al., 2016; Ashbrook et al., 2021). It provides a strong heredity resource that can be used to interpret pathways and regulatory networks using phylogenetic approaches. Some studies have suggested that it is possible to study AD through BXD panel analysis. Previous studies have reported the exploiting inbred lineage properties of AD-BXD groups to explore the molecular mechanisms underlying the early onset of the disease. In addition, several studies have utilized previously generated transcriptomic and phenotypic information from genetically diverse populations of mice to identify the molecular networks directly leading to differences in the cognitive outcomes of different groups (Neuner et al., 2019b; Heuer et al., 2020). We used this powerful resource to explore a strong relationship between amino acids and AD.

In our study, we found that various amino acids are involved in the regulation of AD (data are not shown), among which methionine levels in the midbrain level were interesting. Previous studies mostly focused on the hippocampus (Ren et al., 2021; Xi et al., 2023). For the first time, we reported the relationship between methionine levels in the midbrain and AD. Methionine may promote the progression of AD by regulating the dopaminergic synaptic pathway. The latest evidence of a selective and precocious VTA dopaminergic cell death in a mouse model of AD has implicated the midbrain dopaminergic system in the pathogenesis of AD (Nobili et al., 2017). Furthermore, subcortical dysfunctions, such as reduced dopamine (DA) levels, have also been reported in patients with AD. They lead to psychiatric symptoms, such as apathy and depression, and may be involved in cognitive decline (D'Amelio et al., 2018). Many studies have also shown that the midbrain is involved in learning and memory ability (Schott et al., 2004, 2006; Murty et al., 2011; D'Ardenne et al., 2012; Kafkas and Montaldi, 2015); thus, we speculated that similar to the hippocampus, the midbrain is involved in the development of AD.

**Figure 5:**
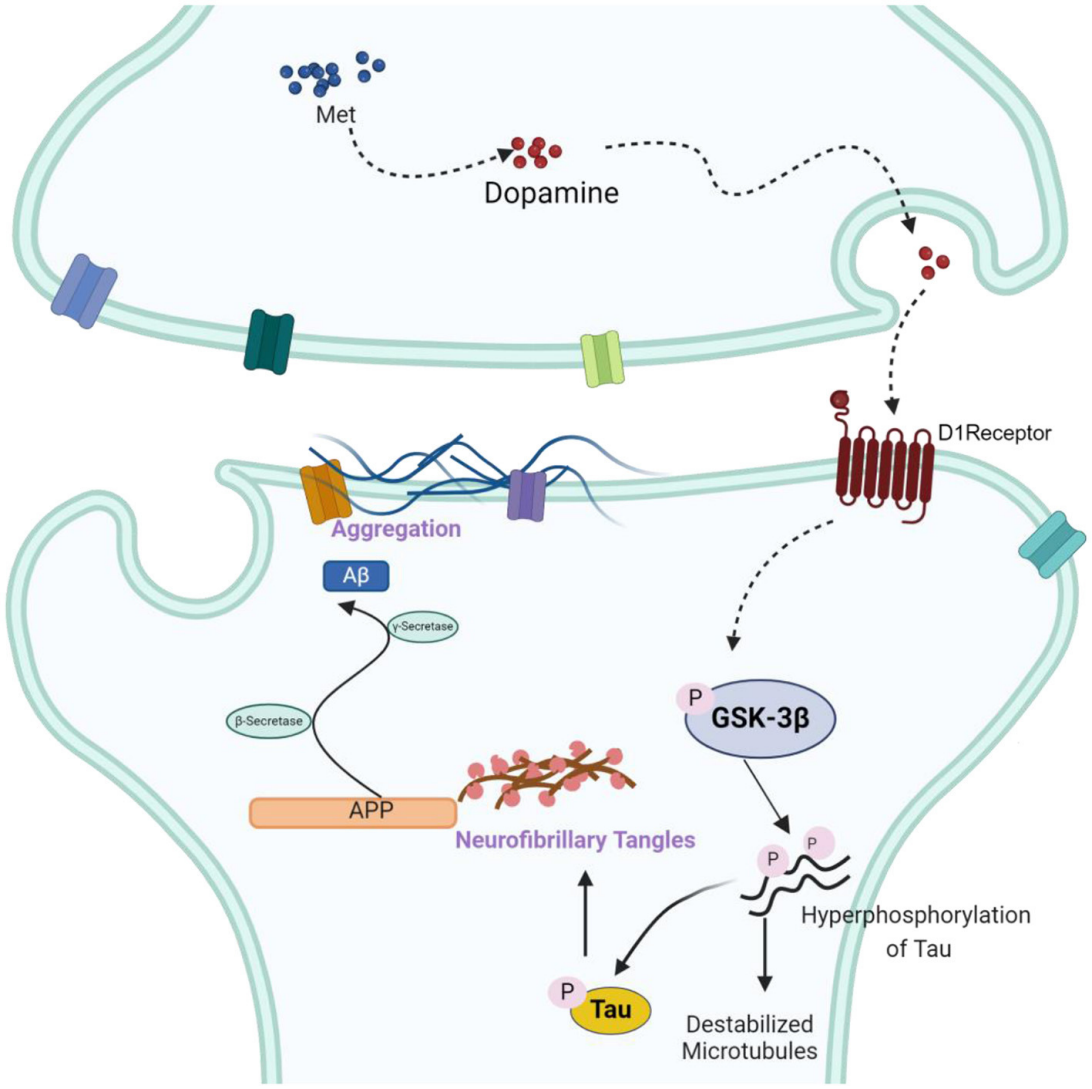
The schematic drawing illustrating the mechanisms underlying the effects of methionine on AD. Accumulation of methionine in the midbrain may increase dopamine secretion, activate dopamine receptors and increase the phosphorylation of GSK-3β, thereby activating GSK-3β, upregulating A-β and p-Tau, and accelerating the progression of AD.

A thorough midbrain gene profiling has shed light on the potential molecular mechanism involved in the association of methionine and AD. Through related gene search and enrichment analysis indicated the enrichment of multiple important neural signaling pathways, such as synaptic vesicle cycle, axon guidance, cholinergic synapse and dopaminergic synapse. The dopaminergic synaptic signaling pathway is one of the most important signal transduction pathways in AD and has a significant role in coordinating neurotransmitters, consolidating memory, and the functioning of synapses (Bamford et al., 2018). Protein-protein interaction network analysis showed that GSK-3β is mostly connected to other nodes in the dopaminergic synaptic pathway. GSK-3β is a downstream mediator of several signaling pathways in the brain, including DA signaling (Li and Gao, 2011; Mahmoodkhani et al., 2022). Thus, methionine may regulate the course of AD by modulating GSK-3β expression. GSK-3β, as an important serine-threonine kinase, is involved in memory consolidation, neurogenesis, synaptic plasticity, long-duration enhancement and inflammation. GSK-3β is abundant in the central nervous system (Lee et al., 2006; Leroy et al., 2007), and regulating GSK-3β activity is considered to be one of the important preventive strategies for neurodegenerative diseases. Through *in vitro* experiments, we found that the phosphorylation level of GSK-3β increased in the presence of high concentrations of methionine (Figure 4C), further validating our hypothesis. When we treated SH-SY5Y cells with an inhibitor of GSK-3β, methionine deficiency or excess no longer altered Aβ and p-Tau levels, demonstrating that GSK-3β is a key downstream protein in methionine-regulated AD (Figure 4D). It has been reported that activation of dopamine D1 receptors can alter synaptic strength and plasticity through GSK-3β activity, which in turn regulates Tau phosphorylation (Lebel et al., 2009). Hence, as shown in the mechanism diagram, we speculated that methionine may affect phosphorylation of Tau and AD progression through dopamine D1 receptor/GSK-3β signaling (Figure 5), but the exact mechanism needs studies.

Still, there are significant limitations to our study. First of all, we only analyzed the correlation between methionine and AD phenotypes in the midbrain, and did not comprehensively analyze relevant data from other brain regions, such as the hippocampus. Secondly, we only preliminarily validated the relevant proteins predicted based on the database, and the more complex regulatory pathways need to be further analyzed. Our study provided a theoretical basis for the role of methionine-restricted diets in improving AD, but we did not conduct animal experiments to further validate the correlation and related pathways. For instance, manipulation of GSK-3β using targeted therapies (e.g., antisense oligonucleotides that inhibit GSK-3β in high-expressing strains or drugs that target the pathway) is needed to study changes in methionine expression and AD progression. Future studies should pay more attention to dietary recommendations for patients with AD.

In summary, using the BXD mouse family as a genetic reference group, we identified a correlation between methionine level in the midbrain and AD. Then we preliminarily explored the regulatory role of methionine in AD mechanism and verified it through *in vitro* experiments. Our study showed that there is a strong correlation between methionine and AD, providing a theoretical basis for the pathogenesis of AD and the prevention and treatment of AD through dietary intervention. Meanwhile, we established a research method to explore the association between metabolites and AD using systems biology. With this method, more metabolites molecules can link to the pathogenesis of AD in the future.

## Data availability statement

The original contributions presented in the study are included in the article/Supplementary material, further inquiries can be directed to the corresponding authors.

## Ethics statement

Ethical approval was not required for the studies on humans and animals in accordance with the local legislation and institutional requirements because only commercially available established cell lines were used.

## Author contributions

CW: Writing – original draft, Investigation, Validation, Visualization. YH: Investigation, Validation, Visualization, Writing – original draft. YLiu: Validation, Writing – original draft. AB: Formal analysis, Writing – review & editing. YLi: Validation, Writing – review & editing. YG: Investigation, Writing – review & editing. FX: Conceptualization, Supervision, Writing – review & editing. CY: Conceptualization, Funding acquisition, Methodology, Resources, Supervision, Writing – review & editing.
